# Long-term effectiveness of behavioural intervention in preschool children with attention deficit hyperactivity disorder in Southeast China – a randomized controlled trial

**DOI:** 10.1186/s12887-021-03046-8

**Published:** 2021-12-10

**Authors:** Xin-xin Huang, Ping Ou, Qin-fang Qian, Yan Huang

**Affiliations:** 1 The ministry of health, Fujian Maternity and Child Health Hospital, Affiliated Hospital of Fujian Medical University, 18 Daoshan Road, Fuzhou, 350001 Fujian Province People’s Republic of China; 2 The child Health Division, Fujian Maternity and Child Health Hospital, Affiliated Hospital of Fujian Medical University, 18 Daoshan Road, Fuzhou, 350001 Fujian Province People’s Republic of China

**Keywords:** Attention deficit hyperactivity disorder, Behavioural intervention, Effectiveness, Randomized controlled trial, Mixed-effects linear model analyses

## Abstract

**Background:**

Attention-deficit hyperactivity disorder (ADHD) is the most common behavioral disorder. Behavioural intervention in preschool children with ADHD is considered effective. This study discussed the long-term effectiveness of behavioural intervention in the context of nondrug therapy.

**Methods:**

The study was a prospective, randomised controlled trial in which 201 preschoolers diagnosed with ADHD who were not receiving any treatment were assigned to two groups from January 2018 to May 2019, 101 were assigned to the conventional group and 100 to the behavioural intervention group. The behavioural intervention group included parental training, behavioural therapy, attention training, relief therapy and game therapy, in addition to the conventional group offerings. Children were evaluated at a baseline, at the end of the 12-month intervention and six months after the intervention. The primary and secondary outcome variables included attention time, the impulse-hyperactivity and hyperactivity index from Conners parent symptom questionnaire (PSQ), full-scale attention quotient (FAQ) and full-scale response control quotient (FRCQ) from integrated visual and auditory comprehensive continuous performance tests. The attention time was observed and recorded by parents, and others were performe(PSQ)d by physicians in the clinic. All statistical analyses were conducted using SPSS V26.0 (IBM), including the descriptive statistics and mixed-effects models and so on.

**Results:**

The participants’ mean age was (66.17±9.00) months in the behavioural group and (67.54±6.22) months in the conventional group .A total of 190 participants completed a follow-up six months after the intervention. The attention time, Conners parent symptom questionnaire (PSQ), full-scale attention quotient (FAQ) and full-scale response control quotient (FRCQ) increased significantly over time, and the behavioural group improvements were higher than those of conventional group. There was a significant main effect of time (pretest/posttest/follow-up) and group on all outcome measures (*t* =-12.549-4.069, *p*<0.05), and a significant interaction of time and group on attention time, impulsivity/hyperactivity, FAQ and FRCQ (*t* =-3.600-3.313, *p*<0.05).

**Conclusion:**

Behavioural intervention can effectively improve behaviour management and relieve symptoms in children with ADHD. These effects lasted at least six months. This study provides a promising approach for improving clinical efficacy with preschool children with ADHD.

## Background

Attention deficit hyperactivity disorder (ADHD) is a commonly diagnosed childhood behavioural disorder [1, 2]. It is characterized by age-inappropriate levels of hyperactivity, impulsivity and inattention, and is often accompanied by comorbid symptoms such as oppositional defiant disorder (ODD) or conduct disorder (CD). Multiple studies show that ADHD symptoms reduce quality of life and are associated with negative academic and socio-occupational outcomes [3–5]. Most children with ADHD tend to have a preschool onset of at least some of their symptoms. Without early treatment, 50-80% of cases may persist into adulthood [6, 7]. Thus, timely interventions in preschool to minimize or eliminate these symptoms are of public health importance.

The purpose of ADHD treatment is to improve the core symptoms and reduce behavioural problems, and the main treatment means are drug therapy and nondrug therapy. Evidence-based treatment for ADHD involves the use of stimulant medication and behavioural interventions [8–10], which have been studied for decades, separately or in combination. However, there is still disagreement among professionals about which one is more effective, as well as how soon to start treatment after diagnosis. Some studies suggested beginning medication immediately and supplementing it with additional medication where necessary [11]. Others recommended beginning with psychobehavioural therapy and adding medication if those treatments were insufficient [12]. Stimulants’ side effects increase with increasing dosage and duration of exposure [13]. For example, the adverse effects of continuous medication on height and weight have always been controversial. Because behavioural interventions may improve outcomes, reduce treatment costs,and minimize side effects [14], it is the first treatment choice for preschool children [15]. The American Academy of Pediatrics recommended behavioural intervention in preschoolers [16]. China's latest guidelines for ADHD diagnosis and treatment also emphasize the use of behavioural interventions in children under the age of 12 [17].

Biofeedback is a therapeutic technique designed to modulate brain function to address neurological and/or psychological symptoms of concern. The standard biofeedback regimen in the treatment of ADHD can be considered a mature treatment with a 32-47% remission rate and sustained effects as assessed after 6-12 months [18].

Behavioural intervention studies on children with ADHD have shown that parental management training effectively reduces children’s ADHD and associated symptoms [4, 5]. Cognitive-behavioral therapy (CBT) interventions can improve the important areas of function for children with ADHD and anxiety, including the severity of ADHD symptoms, according to a randomized controlled trial by Emma [19]. Dose 's telephone-assisted self-help intervention alone for the parents of children with ADHD was proven to be effective in parent-reported externalizing and internalizing child problem behaviour as well as ADHD symptoms and dysfunctional parenting practices [20]. A recent observational study by Manfred also showed that in conventional care settings, children's ADHD symptoms improved after a one-year telephone-assisted parental behaviour intervention and were further improved at the 7-25 months follow-up after completion of the program, with a medium effect size [21]. Such studies suggested that behaviour therapy can improve the prognosis of children with ADHD, and the intervention may have long-term effects.

However, Emma's research objects were 8-12 year old children with ADHD who combined with anxiety; The Dose study aimed at 6-12 pairs of children who still had ADHD and residual dysfunction after methylphenidate treatment; Manfred's study aimed at children between the ages of 6 and 12 and did not set a control group, and 74.3% of the patients were on medication for ADHD before evaluation. We hope to focus on the effect of behavioural therapy on preschool children diagnosed with ADHD without stimulant medication, avoid carrying these problems to school age, or reduce the severity of symptoms at school age. In addition to telephone assisted self-help intervention, we also hope to cooperate with parents to develop a structured strategy for comprehensive behavioural intervention in the context of family, and to know the situation of children at any time through regular follow-up and adjust personalized intervention programs. In addition, large-scale behavioural treatment is complex and expensive, and the availability and effectiveness of treatment may be limited by parents’ education level and their ability to understand and execute the treatment, which hinders the spread of behavioural therapy.

At the research hospital in Southeast China, treatment of children with ADHD was given by paediatricians, child mental health care physicians and psychotherapists. We assessed the 12- month efficacy of cooperation between physicians and parents during behaviour intervention in children with ADHD. We also evaluated whether the improvement in symptoms persisted after 6 months of behavioural intervention. We hope to carry out a controlled trial of behavioural intervention specifically for preschool children to explore the effect of behavioural intervention under the premise of nondrug treatment. We hope that through intensive parental training and guidance, structured interventions can be developed to improve the efficacy of behavioural interventions and carry out scientific evaluation.

## Methods

### Procedure

We aimed to assess the long-term effects of behavioural intervention. At the start of the study, children were randomly assigned to the conventional group or the behavioural group according to the medical card number using SPSS software. The conventional group was subjected to biofeedback and distributed a health education booklet, while the behavioural group received behavioural intervention based on the conventional group. The intervention lasted for one year. Children were followed up for six months after the end of the intervention. If the children or their parents declined to cooperate during the treatment, they were dropped from the study. The physicians evaluated the children at the three time points: before the start of the intervention (T_1_), at the end of the one-year intervention(T_2_) and 6 months after completion of the program(T_3_).

### Trial registration

ChicCTR2100049863 was retrospectively registered on August 9, 2021-Retrospectively registered.

### Participants

Preschoolers with ADHD were recruited from January 2018 to May 2019 to participate in the behavioural intervention study at Fujian Maternity and Child Health Hospital in Southeast China. Inclusion criteria: Children with ADHD who were diagnosed according to the Diagnostic and Statistical Manual of Mental Disorders (5th edition) (DSM-5) by at least two qualified developmental behavioural paediatric psychiatrists. None of the children had received treatment via medication or behavioural intervention. Exclusion criteria: (a) full-scale IQ <75, (b) history of seizures and/or taking medication to prevent seizures, (c) childhood history or concurrent diagnosis with pervasive developmental disorder, schizophrenia or other psychotic disorder, sexual disorder, organic mental disorder or eating disorder, (d) lack of functional impairment, and (e) placement in special education classrooms.

### Biofeedback

An EEG biofeedback system was used to suppress the 4-8 Hz θ wave and strengthen the 12-15 Hz sensorimotor rhythm. Collecting the brain waves of children enabled real-time feedback with a variety of images. Each treatment consisted of five stages, of which the first stage was basic state detection and training target setting. The remaining four stages were feedback therapy stages with 20-30 times/course of treatment, for three courses of treatment [22].

### Behavioural intervention

The following individualized behavioural intervention plans were developed by physicians. (a) Face-to-face parent training: Professional child psychologists set up one-hour, weekly parent classes. At the end of each session, parents received home assignments that were discussed at the beginning of the next session. Where parents failed to fully understand topics discussed before or experienced difficulties with techniques from particular sessions, an extra session was given. (b) Behavioural therapy: Clinicians worked with parents to develop a behavioural training program that included positive reinforcement, elimination and punishment. They developed personalized behaviour training programs and scoring standards. The scores were calculated according to the completion rate and difficulty level of the task. Children were given verbal praise, appropriate material rewards or activity rewards immediately after the appropriate behaviour. When points accumulated to the expected goal, additional rewards were given. For elimination, instead of hitting and scolding children, the parents cancelled or delayed the material reward when the children misbehaved. For punishment, children with impulsive, aggressive behaviour were temporarily isolated or were

immediately removed from the scene. When the children were calm, the reason for punishment was given to help them identify the consequences of poor behaviour. We cooperated with teachers to help track the children's situation in kindergarten, including whether they obey the order of activities, completion of tasks, instructions of teachers, relationships with peers, etc., and with feedback to parents and/or physicians in the form of daily report cards. (c) Attention training: According to the attention time of children at T_1_, the goal of family attention training was set, and children were trained twice a day under the supervision of parents. Adjusted goals regularly according to the children's situation (d) Relief therapy: Children were allowed to speak their dissatisfaction with others so as to release negative emotions. Scheduled activities such as sports and entertainment helped children release excess energy. (e) Game therapy: Role-playing games were designed to stimulate children's enthusiasm and to increase their concentration. The behavioural intervention lasted for one year. Household performance was regularly monitored. Outpatient interviews were conducted weekly for the first two months of intervention, and once every two weeks for the remainder of the period. The outpatient interview was conducted in a face-to-face interview between the paediatricians and the children’s parents. The interview aimed to (a) enhance parents’ and children’s motivation, (b) assess parents' education methods, conduct parent training and guide improvements, (c) check the implementation of the family training program to ensure that they understand the content, (d) adjust the training program to the situation of children at all times. The outpatient interview follow-up lasted for 29.36 minutes on average (SD=2.36). If the child or parent was found to be unwilling or unable to effectively implement the behavioural intervention plan, or if an assessment found that additional medication was needed, he could withdraw from the study at any time. We periodically assessed the child's condition during the follow-up process and decided whether to terminate the intervention.

### Assessment and Instruments

(a) Attention time: Parents were trained on proper ways to track attention span at home. In a quiet environment, children were required to complete drawing, writing or learning assignments based on their age. Parents or physicians observed the children and noted completion times without disturbing them. The clock was stopped whenever the child was distracted or showed redundant movement. This valuation was performed twice a day for 14 consecutive days, and the average value was used for analyses.

(b) Conners parent symptom questionnaire (PSQ): This scale was created by Conners to developed comprehensive checklist for acquiring parental reports of the basic presenting problems [23]. It comprises 48 items and 6 subscales: conduct problems, difficulties in learning, psychosomatic disorders, impulsivity/hyperactivity, anxiety and hyperactivity index [24]. The scale was rated on a 4-point frequency scale ranging from never to frequently. The higher the score is , the more severe the symptoms. The Cronbach’s alpha for PSQ in this study was 0.91. We used impulse-hyperactivity and hyperactivity indices for evaluation. The parents were interviewed by the physicians at the clinic to complete the assessment.

(c) Integrated Visual and Auditory Continuous Performance Test (IVA-CPT): Sustained selective attention was assessed by the IVA-CPT (IVA+Plus, www.braintrain.com) [25], which is a 20-min test with 500 trials of 1 s and 2 s presented in a pseudorandom order to the visual and/or auditor modalities. For this test, the subject clicks the mouse only when a 1 (but not a 2) is seen or heard. The subject’s responses during the test capture abilities of attention, control and focus, both collectively and individually, within the auditory and visual domains. Trained physicians administered the testing to all participants, and responses were converted to age-normed standard scores. In this study, the full-scale attention quotient (FAQ) and full-scale response control quotient (FRCQ) were used to evaluate the participants’ attention deficit and response control, respectively.

### Data collection

Patient information was obtained from the medical records. The research group conducted weekly quality control analyses to eliminate unqualified data.

### Sample size

Based on the pre-experiment, we conclude that a moderate impact amount of 0.5 was detected in the clinically assessed PSQ score (i.e., the difference in average change between the behavioural group and conventional group from start to 12 months), with a two-sided 5% significance level and 80% power. Each group should have at least 85 participants. The number of outpatient visits in the study hospital can ensure a sufficient sample size.

### Randomization

Children were randomized using SPSS 26.0 based on their medical card numbers. The physicians generated the allocation sequence and enrolled participants.

### Blinding

All physicians who performed the intervention signed a memorandum of understanding and agreed not to share intervention materials with nonparticipating families or paediatricians. The physicians who were responsible for the outcome assessment and data analysts were not informed of the treatment grouping. Grouping details were not uncovered until the trial ended unless an emergency occurred.

### Quality control

The physicians who participated in the evaluation were trained to a uniform standard. This training and intervention were described in detail in a parent training class. All anthropometric measurements were performed using the same instrument. We kept a complete list of trial records, including a list of any outcome data to be collected for participants who discontinued the intervention protocols. Plans for data entry, coding, security and storage, including any related processes were performed to promote data quality. At the end of the trial, 10% of children were randomly selected by a third party to review the assessment results. The data monitoring committee consisted of personnel with no competitive interest and reviewed all data records and statistical results at the end of the trial.

### Statistical analyses

Continuous variables were reported as the mean±SD. Categorical variables were shown as numbers (percentages) to describe baseline characteristics. Linear mixed-effects models (LMMs) were used to test changes in outcomes over time, and age was used as a moderating factor. The subjects who quitted the experiment were analysed for baseline data. If applicable, all hypotheses tests were two-tailed. *P*=<0.05 indicated statistical significance. Statistical analyses were done using SPSS 26.0.

## Results

### Study participants

**Fig. 1:**
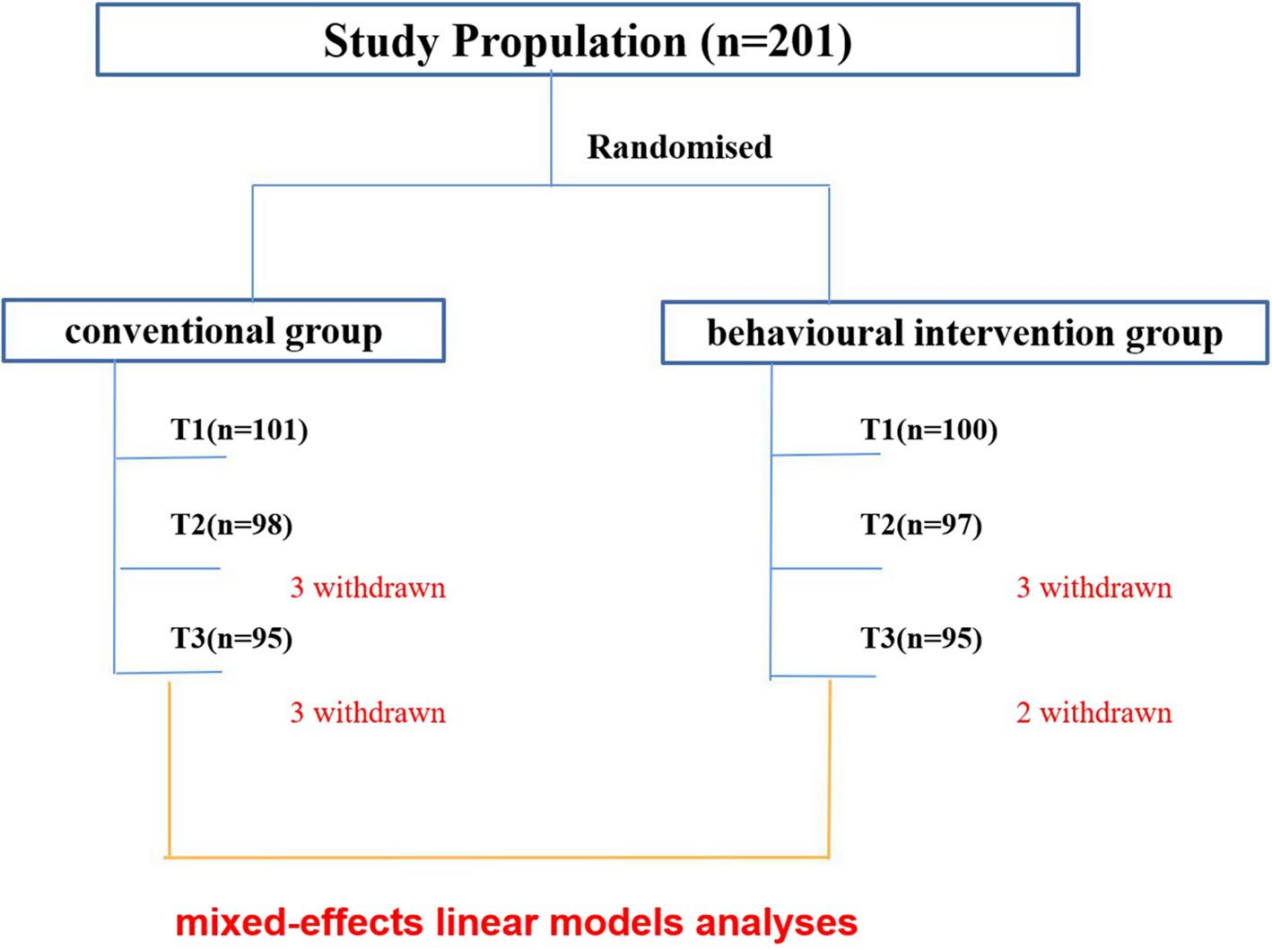
Technical road map. Before intervention, there were 101 and 100 participants in the conventional group and the behavioural intervention group, respectively. At the end of the intervention (one year later), there were 98 participants in the conventional group and 97 in the behavioural intervention group. Six months after the intervention, 95 participants were followed up in both two groups

The participants’ mean age was 66.87 (SD=7.75) months. A total of 126 (70.6%) participants were male, and 59 (29.4%) were female. The total sample contained 201 children, including 100 in the behavioural intervention group and 101 in the conventional group. The duration of intervention was 12 months. A total of 190 participants completed a follow-up six months after the intervention (Fig. 1). At T_1_, the attention time and PSQ scores of the children who left the study were not different from those of the children who completed the study (*P*> 0.05).

Among the 201 participants, The 126 (62.7%) children were diagnosed with inattention type (ADHD-I), 28 (13.9%) with hyperactive-impulsive type (ADHD-H), and 47 (23.4%) with combined type (ADHD-C). Diagnoses were made by the attending paediatrician or adolescent psychiatrist. The groups’ baseline characteristics were comparable (Table 1). There was no gender difference in the IQ distribution between the two groups. In the conventional group, boys were older than girls, but in the behavioural intervention group, there was no difference in age between boys and girls (Table 2).

**Table 1:** Descriptive information of children in both two groups

Items	Behavioural group (*n*=100)	Conventional group (*n*=101)	*t/χ*^*2*^	*P* value
Age [month, *M* (*SD*)]	66.17 (9.00)	67.54 (6.22)	-1.258	0.210
Gender male *n* (%)	67 (67.00%)	75 (74.26%)	1.276	0.259
ADHD- I *n* (%)	57 (57.00%)	69 (68.32%)	2.752	0.253
ADHD-H *n* (%)	16 (16.00%)	12 (11.88%)		
ADHD-C *n* (%)	27 (27.00%)	20 (19.80%)		
Child IQ [*M* (*SD*)]	96.18 (7.91)	94.63 (8.02% )	1.370	0.172
At kindergartenn(%)	96 (96.0%)	100 (99.0%)	----^a^	0.212

^a^ Fisher exact probability

**Table 2:** Age and IQ distribution by gender between the two groups

Group	Items	Boys (*n*=95)	Girls (*n*=95)	*t*	*P* value
Behavioural group	Age [month, *M* (*SD*)]	66.64 (9.45)	65.21 (8.08)	0.745	0.458
	Child IQ [month, *M* (*SD*)]	96.73 (8.26)	95.02 (7.14)	1.067	0.290
Conventional group	Age [month, *M* (*SD*)]	68.40 (6.09)	65.08 (6.05)	2.401	0.018
	Child IQ [month, *M* (*SD*)]	93.34 (8.04)	95.46 (8.05)	0.610	0.543

### Intervention and follow-up

The attention time, impulsivity hyperactivity, hyperactivity index, FAQ and FRCQ scores of the two groups of children at different times were showed in Table 3. At T_1_, the attention time of the behavioural group was 5.02, while that of the conventional group was 5.24, which were 8.68 and 7.55 at T_2_ and 9.13 and 7.80 at T_3,_ respectively. At T_1_, the impulsivity hyperactivity of the behavioural group was 1.85, while that of the conventional group was 1.74, which were 1.29 and 1.55 at T_2_ and 1.19 and 1.45 at T_3,_ respectively. At T_1_, the hyperactivity index of the behavioural group was 2.08, while that of the conventional group was 2.22, which were 1.68 and 1.95 at T_2_ and 1.58 and 1.84 at T_3,_ respectively. Differences in attention time, impulsivity hyperactivity, hyperactivity index, FAQ and FRCQ were examined for changes in values-values, F values were determined by mixed-effects linear model analyses using time, group and time*group as fixed effects, subjects as random effects, and gender and age as the covariates. There was a significant main effect of time (pretest/posttest/follow-up) and group on all outcome measures (*t* =-12.549-4.069, *p*<0.05), and a significant interaction of time and group was found on attention time, impulsivity hyperactivity, FAQ and FRCQ (*t* =-3.600-3.313, *p*<0.05), which were shown in Table 4.

**Table 3:** The Impulsivity hyperactivity, hyperactivity index,attention time, FAQ and FRCQ of two groups of children with ADHD at different times

Items	Behaviour group (*n*=95)			Conventional group (*n*=95)		
	Pre-intervention (T_1_)	Post-intervention (T_2_)	Follow-up (T_3_)	Pre-intervention (T_1_)	Post-intervention (T_2_)	Follow-up (T_3_)
Attention time	5.02 (1.62)	8.68 (1.94)	9.13 (2.14)	5.24 (2.23)	7.55 (2.16)	7.80 (2.30)
Impulsivity hyperactivity	1.85 (0.65)	1.29 (0.30)	1.19 (034)	1.74 (0.65)	1.55 (0.60)	1.45 (0.48)
Hyperactivity index	2.08 (0.54)	1.68 (0.53)	1.58 (0.53)	2.22 (0.41)	1.95 (0.40)	1.84 (0.41)
FAQ	77.54 (11.76)	92.41 (8.03)	93.83 (8.09)	76.30 (8.92)	88.11 (6.46)	88.37 (6.42)
FRCQ	70.69 (5.27)	94.60 (9.76)	99.92 (6.69)	71.69 (8.46)	94.55 (10.37)	96.29 (9.20)

**Table 4:** The estimates of fixed effects of the impulsivity hyperactivity, hyperactivity index,attention time, FAQ and FRCQ

Items		*t*	*p*	95% CI	
Attention time	group	4.069	0.000	0.677	1.952
	time	-7.475	0.000	-3.446	-2.011
	group*time	-3.600	0.000	-2.393	-0.702
Impulsivity hyperactivity	group	-4.069	0.000	-0.368	-0.128
	time	4.540	0.000	0.237	0.599
	group*time	3.313	0.001	0.150	0.589
Hyperactivity index	group	-4.005	0.000	-0.408	-0.139
	time	3.608	0.000	0.136	0.463
	group*time	1.402	0.162	-0.055	0.329
FAQ	group	3.914	0.000	2.498	7.612
	time	-3.247	0.001	-11.767	-2.889
	group*time	-2.031	0.044	-9.170	-0.136
FRCQ	group	2.492	0.014	0.757	6.615
	time	-12.549	0.000	-31.013	-22.606
	group*time	-2.392	0.018	-8.415	-0.814

## Discussion

Given recent studies on the lack of long-term efficacy and tolerance to extended use of central nervous system stimulants, it is necessary to limit life-long dosing [26], especially for preschoolers. Here, we show that adding behavioural interventions to traditional treatments effectively improves behavioural challenges and reduces ADHD symptoms in children. We experimentally examined the long-term effectiveness of behavioural intervention on children with ADHD and found persistent improvements in child behaviour during the course of intervention and follow-up. Children with ADHD were followed up for 18 months and completed the attention time, PSQ and IVA-CPT at the baseline (T_1_) , 12 months (T_2_) and 6 months after the intervention (T_3_) .During intervention and follow-up, children in both groups had longer attention spans, lower PSQ scores (impulsivity hyperactivity and hyperactivity index) and increased FAQ and FRCR. The behavioural group improved significantly compared with the conventional group. These improvements remained stable over a 6-month period, which indicated that gains achieved at posttest remained stable over time.

In this study, we show that adding behavioural interventions to traditional treatments effectively improves behaviour and reduces ADHD symptoms in children.

Because the PSQ score outcome measures were parent-reported, it was possible that the test data were influenced by socially desirable response bias. In recent years, scholars have focused on developing objective ADHD assessment tools [27]. Past studies showed that IVA-CPT accurately identified children with ADHD [28]. Thus, it was used for auxiliary ADHD diagnosis and to evaluate treatment efficacy. Different from the existing studies, in addition to IVA-CPT, we also used children's attention time measured under strict quality control as an objective indicator. The evaluation of these objective indicators provided evidence for the effectiveness of behavioural therapy.

The reported behavioural therapy included parent training programmes, instruction manuals for parents and regular teleconferencing by professionals [21]. Our research design was slightly different. Our interventions included on-site parent training, behavioural therapy programmes and regular one-on-one outpatient interviews and evaluations. As one of the baseline data, attention time was one of the bases for formulating attention training in behavioural intervention. These advantages lay in strengthening the interactions among physicians, parents and children, timely identification of inappropriate family behaviour interventions, helping parents understand children's attention, judging the effect of behaviour interventions, observing and collecting changes in children's symptoms in a timely manner, adjusting personalized behavioural therapy programs, providing personalized counselling and treatment strategies for parents, and improving compliance.

The results of a randomized controlled study [29] of IVA-CPT tests in children with ADHD showed that behavioural therapy was more effective in response control and attention improvement than biofeedback and pharmacological therapy. Recent studies using within-subject and RCT designs to evaluate various medication doses in various combinations with behavioural treatments [30], have consistently shown that intensive behaviour modification achieves acute effects similar to relatively high medication doses. Those studies provided evidence for the effectiveness of behavioural interventions that reduce behavioural problems in children, as well as attention loss, impulsivity and hyperactivity issues, consistent with our findings.

behavioural management training for parents aims at modifying parenting behaviour and enhancing their skills to improve ADHD symptoms in children. The evaluation showed that parent management training interventions were effective in reducing children’s ADHD and related symptoms [5]. It was shown that even brief parenting intervention can reduce child externalizing behaviours and dysfunctional parenting strategies. The changes were maintained at 6-month and 1-year assessments [31, 32]. A randomized controlled trial found that gains from self-directed behavioural family intervention can even remain 3 years after its completion [33]. These reports were consistent with our findings that overall behavioural problems continued to improve from assessment to follow-up and remained stable for 6 months. However, our study was followed up only 6 months after the intervention, and future studies should have longer follow-up times to determine long-term outcomes.

Inadequate access to interventions by education, health and mental health professionals, and cross-system collaboration are challenges for behavioural therapy. The US developed the collaborative life skills (CLS) program [34], which was implemented in schools by school mental health providers (SMHP) and offered to students with ADHD presentations. Students assigned to CLS showed significantly greater improvement at follow-up. The study showed that CLS improved ADHD and ODD symptoms, as well as organizational skills and academic competence, which were maintained into the next school year.

This study confirmed the efficacy of strict behaviour management by parents under the guidance of physicians for children with ADHD. However, for children starting kindergarten, behaviour management in school is equally important. In the future we plan to expand the scope of behavioural intervention, and physician-teacher-parent-children integrated intervention strategies should be established to perform a closed-loop management. Structured form management trains teachers in the behaviour management procedures in schools to encourage children with ADHD to abide by rules, stay on tasks, and be productive. In addition to punishment and reinforcement, approaches such as daily report cards, token economy, time out from positive reinforcement, and their effective implementation will be taught. Further high-quality multicentre studies will be carried out to evaluate the long-term outcomes of tripartite behavioural therapy by clinicians, parents and school teachers and to standardize the behaviour management mode of ADHD.

## Strengths and Limitations

Due to the close interaction with parents, the rate of loss to follow-up in this study was very low. We used strictly measured attention time as an objective indicator with good reliability. Therefore, it can help parents observe the performance of children at any time, as one of the bases of family behaviour management, and improve the compliance of parents. The sample size of this study was relatively large to provide sufficient statistical power when detecting associations, and mixed-effects models were used to correct for possible influencing factors.

There were some limitations in our study. First, due to the long duration of the intervention (one year), the inclusion of a completely blank control was ethically disallowed. However, we used the conventional approach (biofeedback and health education) as the control group and observed the advantage of adding behavioural intervention to the conventional approach. Second, our data were collected at one hospital, and the parents involved in the study may have better education or higher economic status than the general population, increasing their health care awareness, so the extension in other populations remains to be studied.

## Conclusions

This study provided a comprehensive behavioural intervention based on the traditional therapy that promoted the improvement of ADHD symptoms in preschool children. Our data indicated that once skills were established through a training intervention, they became routine and improved the effectiveness of behavioural therapy,which might help to facilitate the long-term clinical outcome and quality of life of children with ADHD. Therefore, we should formulate a structured behaviour management plan to regulate ADHD in the future.

## Data Availability

Data are available upon reasonable request. The authors are not authorized to share unauthorized data with a third party. However, data for statistical analysis are available from the corresponding author on reasonable request.

## Abbreviations

ADHD: Attention deficit hyperactivity disorder
ODD: Oppositional defiant disorder
CD: Conduct disorder
